# Uterine Rupture Following Non-Operative Vaginal Delivery: A Close Save of Delayed Presentation With Hemoperitoneum to a Rural Tertiary Care Hospital

**DOI:** 10.7759/cureus.21076

**Published:** 2022-01-10

**Authors:** Surekha Tayade, Arzoo Chadha, Smriti Khandelwal, Nidhi Makhija, Hard Tilva, Sparsh Madaan

**Affiliations:** 1 Department of Obstetrics and Gynaecology, Jawaharlal Nehru Medical College, Datta Meghe Institute of Medical Sciences, Wardha, IND

**Keywords:** maternal near miss, non-operative vaginal delivery, rural tertiary hospital, hemoperitoneum, uterine rupture

## Abstract

Hemoperitoneum as a result of uterine rupture in a previously unscarred uterus is a rare entity to encounter and a potentially life-threatening condition. Ruptures can occur in a scarred uterus either spontaneously, due to operative manipulations, or with the use of uterotonic medications. In an unscarred uterus, spontaneous ruptures are known with high parity, use of oxytocin, and prolonged, neglected labor. Ruptures can be silent with no symptoms resulting in a delay in diagnosis and a near-miss situation. Here, we report the case of a 25-year-old young female who was referred to our tertiary care hospital in rural central India six hours after full-term vaginal delivery, which was followed by pain in the lower abdomen. She had no history of cesarean section, laparoscopic procedures, or surgical termination of pregnancy, which would have predisposed her uterus to rupture. She was severely pale on arrival, and a contrast-enhanced computerized tomography scan revealed rupture of the left side of the uterus with hemoperitoneum and a large pelvic hematoma. Because the patient was in hemorrhagic shock, she was immediately taken for laparotomy with simultaneous resuscitative measures and blood transfusion on flow. Extensive uterine rupture, extending through the cervix to the round ligament of the left side involving the left lateral uterine wall, with active bleeding from the site of the defect was confirmed. The hematoma was 10 × 10 cm in size and was evacuated, following which peripartum hysterectomy was done. The left ureter was traced and safeguarded while applying the clamp on Mackenrodt’s ligament. The patient recovered completely following the procedure. She was discharged on day 13 in stable condition. She is currently doing well on follow-up and is a good example of a maternal near miss. In this report, we emphasize that, even in the absence of any obvious risk factor, uterine rupture can occur during labor, and monitoring the vitals of patients in the immediate postpartum period is essential to detect and promptly manage this serious condition for preventing maternal mortality.

## Introduction

Rupture of a healthy, unscarred uterus is a rare but severe life-threatening condition leading to increased maternal mortality. Population-based studies have not included uterine rupture as many cases remain asymptomatic and due to the lack of a definitive clinical presentation [1]. According to the World Health Organization, the incidence of uterine rupture is 5.3 per 10,000 deliveries. This condition is catastrophic and associated with high maternofetal mortality and morbidity. Maternal mortality has been reported to range from 1% to 13% [2].

Although several risk factors have been identified for uterine rupture, a history of cesarean section is the most important risk factor, especially in developed countries where the incidence of cesarean section is relatively higher than in developing countries [3]. A rupture in the unscarred uterus is considerably rarer. High parity, prolonged and obstructed labor, uterine manipulation, operative vaginal deliveries, and injudicious use of uterotonics are some of the risk factors.

Patients may present with maternal tachycardia and hypotension associated with pain in the abdomen. However, oftentimes the clinical picture is non-specific and a high index of suspicion is required to promptly diagnose and manage the condition for preventing maternal mortality [4]. Placental factors such as placenta previa and drugs such as the use of prostaglandin or oxytocin for induction or augmentation of labor may also predispose an individual to develop uterine rupture. Delay in diagnosis, poor transport facilities, social factors such as poverty and lack of awareness, and anemia may lead to poor outcomes [5].

## Case presentation

A 25-year-old female was referred to a rural tertiary care hospital in central India by a private practitioner, practicing at a distance of 10 km from the hospital. She was a second gravida with previous vaginal delivery. Her chief complaint was pain in the lower abdomen, located on the left iliac region, since the delivery of the child six hours back. She had previously delivered a female child weighing 3.5 kg six years ago, which was a normal full-term vaginal delivery. During the current pregnancy, she had no unusual complaints. She was admitted in active labor and progressed normally without the administration of uterotonics. She delivered a 3.7 kg male child, without any untoward event or the use of any operative procedure. Bleeding was average after childbirth; however, half an hour later, she started to complain of steadily increasing pain in the lower abdomen. She also had three to four episodes of vomiting, and her systolic blood pressure was 90 mmHg at the time. The treating obstetrician resuscitated the patient with intravenous (IV) fluids, gave her injectable antiemetic, and kept her under observation. However, the pain was persistent over the next few hours. A bedside ultrasound was performed to rule out any hemorrhagic event. With the suspicion of pelvic hematoma, the patient was referred to our hospital.

**Figure 1:**
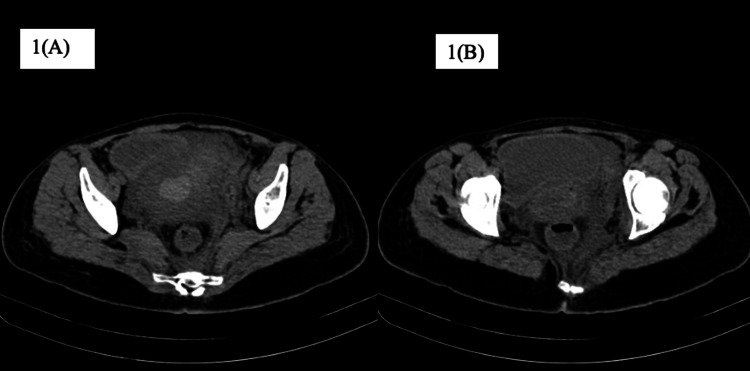
A: Contrast-enhanced CT showing hemoperitoneum on both sides of the uterus. B: Contrast-enhanced CT showing air pockets inside the uterus. CT: computerized tomography

**Figure 2:**
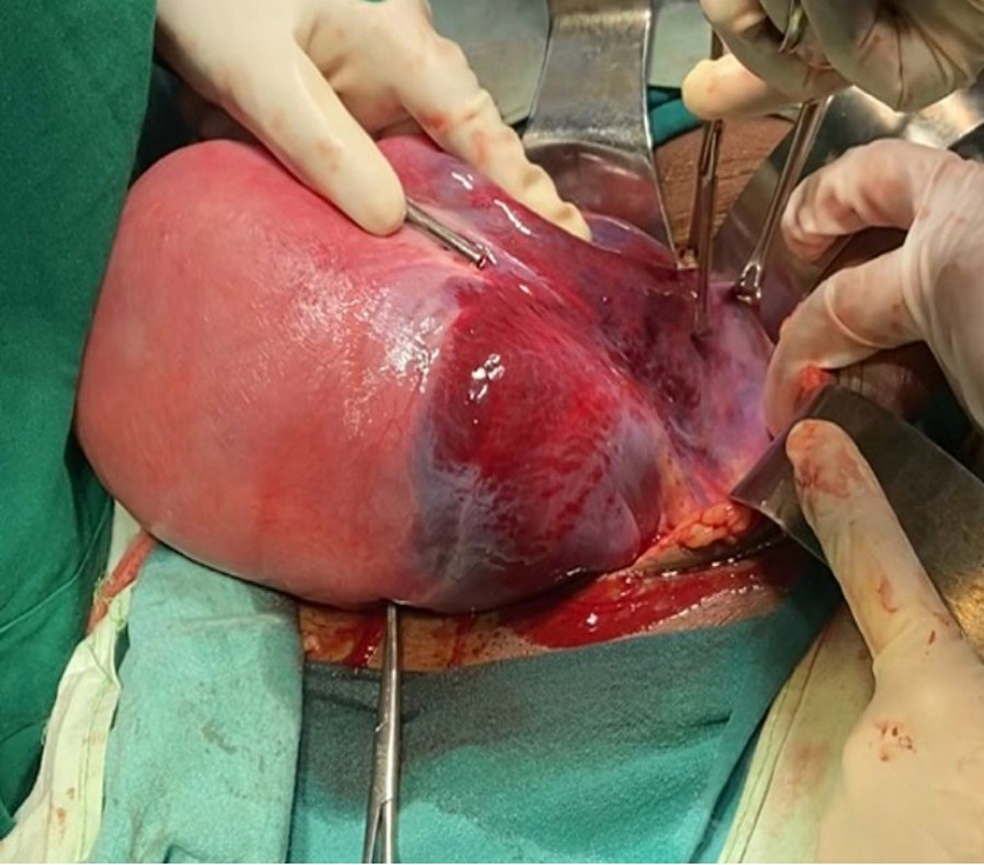
The reddish discoloration extending up to the left round ligament, indicating the hemorrhagic event. The rupture was visible after opening the anterior fold of the peritoneum.

**Figure 3:**
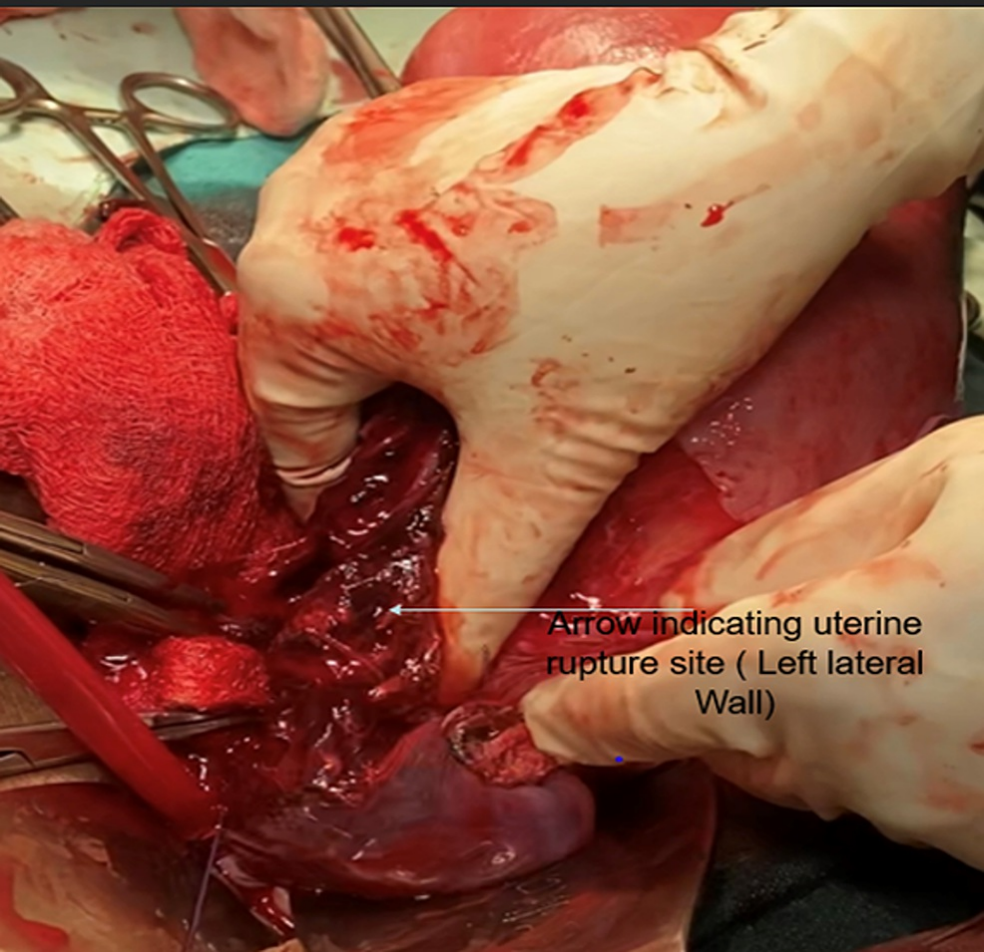
Rupture of the ovary on the left side involving the left uterine wall.

On arrival, the patient’s pulse was 110 beats per minute, regular, blood pressure was 80/50 mmHg in the right arm in a supine position. In addition, pallor and tenderness over the abdomen were also noted. A vague mass of 6 × 6 cm in size was palpable in the left iliac region, and a contracted uterus was palpated, up to the umbilicus, pushed to the other side. Laboratory investigations revealed hemoglobin of 6.1 g/dL with a mean corpuscular volume of 82 fl. The rest of the laboratory investigations were within normal limits. Initial resuscitation of the patient was done, and a contrast-enhanced computerized tomography (CT) scan was obtained. The CT scan revealed uterine rupture of the left side with a large adjacent hematoma of approximately 8 × 8 cm in size (Figure 1A). Moreover, there was blood density collection in the endometrial cavity and peritoneal cavity outside the uterus but adjacent to it on the left side. There was discontinuity in the uterine wall on the left lateral side, and multiple air density foci suggested perforation of the uterus with hematoma in the peritoneal cavity (Figure 1B). The blood density collection measured 12.3 × 6.7 × 6.4 cm in size. The patient was shifted immediately for emergency laparotomy and exploration with simultaneous blood transfusion on flow. On opening the abdomen, the uterus was seen pushed to the right site and a reddish discolored mass was visible on the left (Figure 2). On opening the anterior leaf of the peritoneum, a large clot of 8 × 8 cm in size was observed, which was evacuated to reveal a rent in the left uterine wall extending up to the cervix below and the left round ligament above (Figure 3). The decision of hysterectomy was taken and the uterus was exteriorized. Kelly clamps were placed on bilateral round ligament along with the cornual structures and were clamped, cut, and ligated. The uterus was held from both sides using Metzenbaum scissors. The ureter on the left side was traced and safeguarded while dissection was carried out in the area. The uterine arteries were skeletonized bilaterally and clamped and ligated. Bilateral uterosacral ligaments and Mackenrodt’s ligament were clamped and ligated. Bilateral Mackenrodt’s ligament was also similarly managed, and with careful precision, hysterectomy was performed. Both ovaries were preserved. Two units of packed red cells were transfused postoperatively. The patient recovered gradually, with her blood pressure improving to 100/60 mmHg. She was managed with empirical antibiotics and physiotherapy along with IV fluids postoperatively. She was discharged in a stable condition on day 13 of admission.

## Discussion

Uterine rupture in a healthy unscarred uterus has an incidence of 0.006-0.012% [6]. Clinical presentation can be in the form of abnormal fetal heart rate, with the most common abnormalities of pain in the abdomen and bleeding per vaginum along with altered contraction of the uterus. Rare presentations include shock hematuria and pain in the shoulder tip or scar. The most common of the above-mentioned presentations are pain in the abdomen along with fetal bradycardia. In case of a gravid or immediate postpartum woman presenting with abdominal pain, shock, hypotension, fetal distress, and vaginal bleeding, rupture of the uterus should be suspected [7]. Earlier it was believed that the loss of uterine contraction is characteristic to diagnose rupture of the uterus; however, it is now known that normal contraction or hyperstimulation of the uterus can also be associated with rupture of the uterus.

Important differential diagnoses of uterine rupture include abruption of placenta, placenta previa, inversion of the uterus, tear of the cervix or vagina, coagulopathy, atonic uterus, and rupture of the uterine artery [8-10]. During pregnancy, clinical features of hemoperitoneum can mimic abruption of the placenta, and endometriosis can lead to erosion of blood vessels leading to severe hemorrhage [11].

In the present case, there was an absence of any prior risk factor for rupture of the uterus. Previous injury to the uterus was unlikely to be the cause of uterine injury as the previous pregnancy was uneventful with normal full-term vaginal delivery and no history of previous uterine surgery. However, an undiagnosed mild uterine injury during the previous pregnancy could have been the cause of the uterine rupture during the present pregnancy. There was no history suggesting endometriosis in our patient with no evidence of endometriosis found during the laparotomy. Adenomyosis was also ruled out as the potential cause due to the absence of characteristic features on histopathological examination of the removed uterus [12]. There was no evidence of abnormal placentation on routine ultrasonography performed throughout pregnancy, and the obstetrics team that had conducted the delivery had reported a normal placenta which was delivered 30 minutes after the delivery. The patient was healthy and had no family history of muscular diseases.

In rupture of a previously unscarred uterus, prompt diagnosis and management help prevent maternal mortality. An abdominal CT scan was extremely useful in diagnosing uterine rupture, and laparotomy followed by hysterectomy ensured control of the bleeding [13,14]. Hemorrhagic shock was managed successfully through blood transfusion, and the patient did not require any inotropic support [15].

Difficulties faced in the diagnosis of uterine rupture in our case can be linked to various factors. Uterine rupture is rare, and in patients with non-suggestive obstetric history, it is difficult to pinpoint rupture of the uterus as the diagnosis [16]. In such patients with atypical history, the diagnosis of uterine rupture might be delayed significantly or may be established only during laparotomy with macroscopic evidence. Because this can result in maternal and fetal mortality, treating clinicians should be aware of such atypical presentations of uterine rupture [17].

## Conclusions

Uterine rupture should be considered as an important differential diagnosis in women presenting with hemoperitoneum even when typical risk factors such as previous cesarean section are not present in the obstetric history of the patient. Prompt diagnosis and management of uterine rupture in such cases can prevent maternal mortality, especially in rural areas where the presentation of uterine rupture might be delayed.

## References

[1] Sinha M, Gupta R, Gupta P, Rani R, Kaur R, Singh R (2016). Uterine rupture: a seven year review at a tertiary care hospital in New Delhi, India. Indian J Community Med.

[2] Hofmeyr GJ, Say L, Gülmezoglu AM (2005). WHO systematic review of maternal mortality and morbidity: the prevalence of uterine rupture. BJOG.

[3] Fitzpatrick KE, Kurinczuk JJ, Alfirevic Z, Spark P, Brocklehurst P, Knight M (2012). Uterine rupture by intended mode of delivery in the UK: a national case-control study. PLoS Med.

[4] Abdalla N, Reinholz-Jaskolska M, Bachanek M, Cendrowski K, Stanczak R, Sawicki W (2015). Hemoperitoneum in a patient with spontaneous rupture of the posterior wall of an unscarred uterus in the second trimester of pregnancy. BMC Res Notes.

[5] Catanzarite V, Cousins L, Dowling D, Daneshmand S (2006). Oxytocin-associated rupture of an unscarred uterus in a primigravida. Obstet Gynecol.

[6] Vernekar M, Rajib R (2016). Unscarred uterine rupture: a retrospective analysis. J Obstet Gynaecol India.

[7] Salama S, Nizard J, Camus E, Ville Y (2009). Spontaneous haemoperitoneum after the second trimester of pregnancy. Diagnosis and management. Eur J Obstet Gynecol Reprod Biol.

[8] Purdie FR, Nieto JM, Summerson DJ, Livermore WE (1983). Rupture of the uterus with DIC. Ann Emerg Med.

[9] Vyjayanthi S, Rajesh U, Bloomfield TH (2002). Haemoperitoneum due to placenta percreta in the third trimester mimicking placental abruption. J Obstet Gynaecol.

[10] da Silva CM, Luz R, Almeida M, Pedro D, Paredes B, Branco R, Pereira A (2020). Hemoperitoneum during pregnancy: a rare case of spontaneous rupture of the uterine artery. Case Rep Obstet Gynecol.

[11] Kim BH, Park SN, Kim BR (2020). Endometriosis-induced massive hemoperitoneum misdiagnosed as ruptured ectopic pregnancy: a case report. J Med Case Rep.

[12] Li X, Li C, Sun M, Li H, Cao Y, Wei Z (2021). Spontaneous unscarred uterine rupture in a twin pregnancy complicated by adenomyosis: a case report. Medicine (Baltimore).

[13] Bhoil R, Surya M, Mistry KA (2016). CT diagnosis of spontaneous uterine rupture at term, sonographic appearance of which was confused with placenta praevia. Ann Saudi Med.

[14] Şahin O, Tahtabaşı M (2020). Uterine rupture following vaginal birth after caesarean section (VBAC): CT findings and clinical course in delayed cases. Jinekoloji.

[15] Egbe TO, Halle-Ekane GE, Tchente CN, Nyemb JE, Belley-Priso E (2016). Management of uterine rupture: a case report and review of the literature. BMC Res Notes.

[16] Halassy SD, Eastwood J, Prezzato J (2019). Uterine rupture in a gravid, unscarred uterus: a case report. Case Rep Womens Health.

[17] Kumba C, Graignic A, Philippe A (2017). Complete uterine rupture: a case report. J Anesth Crit Care.

